# The Month of May in India

**Published:** 1890-05-24

**Authors:** 


### The Month of May in India.

During the month of May and part of June in India there is seldom a fleecy cloud upon the sky to screen the inhabitants from the intensity of the vertical solar rays. A tide of flickering exhalations, or simmering air, is seen steaming from the plains, assuming the fantastic forms of mirage—resembling lakes where there is no water, and trees and forests where no vegetation exists. The ground is parched, rent, and riven, as if baked over a volcano. The earth, with the exception of some few trees which keep their foliage, is stripped of all herbage, as if a flood of lava had flowed over it; and water, except in large rivers and lakes and deep wells, is dried up. Hot winds blow with the ardour of a blast from a furnace, and are so laden with dust that it insinuates itself into every crevice, and covers every article of furniture. The nostrils become encrusted, the ears filled, the eyes irritated sometimes into ophthalmia, and the lungs and air passages into bronchial irritation. In some districts, the Punjaub for instance, the dust is something incredible. For days the sun is obscured, and an elephant might pass a short distance away without being seen. Often the wind lulls at night, when radiation from the heated house is more distressing in the still air. Then beds are carried out of doors under the open sky, and people endeavour to sleep with no other covering than the floating dust, where the starry firmament should be. One now becomes sensible of increased frequency of respiration to compensate for the lessened oxygen in an equal bulk of the rarified air, and often heat asphyxia ensues. In other localities, where hot winds do not blow, the air is moist and saturated. The slightest exertion fatigues, streams of perspiration trickle down the body, and at night the bed is wet from a similar cause. But when the rains set in a change appears. The rebellious dust becomes mud. Rivers are full to overflow. Every ditch is a canal. Every hole a pool of water. Every hollow a lake, and the whole face of the country changes as if by magic from a dust-brown to a vivid green, while dry atmosphere becomes moist, and moist atmosphere becomes over-saturated. It is then that diarrhœas, dysenteries, and other abdominal diseases abound.

